# The impact of perioperative fasting on metabolic status in elective caesarean section patients

**DOI:** 10.4102/safp.v68i1.6303

**Published:** 2026-05-13

**Authors:** Sivesh Seevnarain, David G. Bishop

**Affiliations:** 1 Department of Anaesthesiology and Critical Care, University of KwaZulu-Natal, Pietermaritzburg, South Africa

**Keywords:** obstetric anaesthesia, acid and base disturbances, starvation, ketoacidosis, hypoglycaemia

## Abstract

**Background:**

Obstetric patients exhibit ‘accelerated starvation’, increasing the risk of ketoacidosis. Fasting guidelines aim to reduce pulmonary aspiration, but prolonged preoperative starvation may cause metabolic disturbances. This study aimed to assess the relationship between fasting duration and the incidence of metabolic disturbances.

**Methods:**

We conducted a prospective observational study of patients undergoing elective caesarean section at Harry Gwala Regional Hospital. Fasting duration was recorded via questionnaire. Preoperative venous bicarbonate, base excess, glucose and ketone levels were measured to determine the incidence of metabolic disturbances before anaesthesia.

**Results:**

We recruited 107 patients. Of which, 30% were acidotic (base excess less than −3). The acidotic group had a longer starvation time than the non-acidotic group (mean starvation time 14.8 h [standard deviation [s.d.] 2.68, 95% confidence interval [CI] 13.8–15.7] versus 12.75 h [s.d. 3.18, 95% CI 11.8–13.2]; *p* < 0.01). Ketosis occurred in 32% of patients and was associated with longer starvation times (*p* < 0.01).

**Conclusion:**

Starvation periods exceeded recommendations and were associated with metabolic disturbances, including acidosis, hypoglycaemia and elevated ketones in elective caesarean patients. Adherence to fasting guidelines should be reinforced, and patients with extended starvation require further assessment. Future research should focus on optimising fasting durations and improving compliance with established guidelines.

**Contribution:**

This study demonstrates the metabolic impact of extended preoperative fasting periods in elective caesarean sections. Findings reinforce the need for quality improvement programmes to reduce prolonged fasting periods in the perioperative period.

## Introduction

Pregnant patients are at increased risk for ketoacidosis, a condition marked by significant acidosis due to insufficient glucose availability and insulin secretion. This is exacerbated during pregnancy because of an exaggerated response to fasting, often referred to as ‘accelerated starvation’.^1^ While healthy non-pregnant individuals can endure up to 14 days of starvation before experiencing severe ketoacidosis, pregnant women can show significant ketone levels after just 12 h of fasting.^1,2,3^ This is largely due to a combination of low insulin and elevated levels of placental hormones such as glucagon, cortisol and human placental lactogen, which counteract insulin’s effects.^4^

Preoperative fasting protocols are standard practice in anaesthesia before elective surgery. The 2022 South African Society of Anaesthesiologists (SASA) practice guidelines recommend fasting times of at least 6 h for solid food and 2 h for clear fluids. However, numerous studies have demonstrated that obstetric patients frequently experience extended periods of fasting.^5,6,7^ In a South African study, Morgan et al. confirmed the occurrence of extended fasting periods, with many patients experiencing repeated prolonged fasting due to surgical cancellations and system-related factors.^8^ These findings highlight a clinically relevant and potentially modifiable risk factor in perioperative obstetric care.

The presence of metabolic acidosis in the perioperative phase may result in increased maternal and foetal morbidity. Maternal effects include hypotension, a decreased response to catecholamines, reduced myocardial contractility and an increased risk of arrhythmias, which may contribute to perioperative cardiovascular instability, particularly with neuraxial anaesthesia. Uteroplacental perfusion may be compromised secondary to maternal hypotension. Foetal consequences may include low Apgar scores, poor respiratory effort, increased risk of intensive care admission and foetal acidosis.^9,10^

Several case reports describe third-trimester patients presenting with severe high anion gap metabolic acidosis attributed to euglycaemic starvation ketoacidosis following prolonged fasting.^4,9,11,12,13,14,15,16^ While these reports highlight the potential severity of this condition, reliance on case studies prevents accurate estimation of its incidence among patients undergoing standard fasting protocols. Furthermore, the degree of ketosis and the frequency of clinically significant metabolic disturbances in this population remain unclear.

There is a lack of prospective data evaluating the incidence of metabolic derangements, including acidosis, ketosis and hypoglycaemia in obstetric patients subjected to routine preoperative fasting practices. This represents an important knowledge gap, particularly in settings where fasting durations may be prolonged due to constraints within the healthcare system. A better understanding of these metabolic changes is necessary to inform safe fasting practices and optimise perioperative care.

This study is based on the physiological interaction between pregnancy-induced insulin resistance and fasting-induced carbohydrate depletion, which promotes ketosis and metabolic acidosis. The severity of these metabolic disturbances is likely influenced by fasting duration. These maternal metabolic changes may, in turn, affect haemodynamic stability and uteroplacental perfusion, thereby influencing foetal outcomes.

This study aimed to determine the incidence of metabolic disturbances in patients undergoing elective caesarean section following standard preoperative fasting protocols at a South African regional hospital. The objectives were to determine the incidence of metabolic acidosis, quantify the degree of ketosis, assess perioperative glucose levels and the incidence of hypoglycaemia and to evaluate the relationship between fasting duration and metabolic derangements.

## Research methods and design

### Study design

We conducted a prospective observational study of patients scheduled for elective caesarean section at Harry Gwala Regional Hospital. We aimed to identify the incidence of significant metabolic dysfunction by examining bicarbonate, base excess and glucose levels through venous blood gas analysis. We also assessed blood ketone levels using a blood ketone monitor (Abbott Freestyle Optium^®^ Neometer, Chicago, Illinois, United States).

### Setting

The study was conducted at Harry Gwala Regional Hospital, where elective caesarean sections are performed in a district or regional-level obstetric service within the South African public health sector.

### Study population and sampling strategy

The study cohort comprised patients who were planned for elective, full-term caesarean section. All patients included were fasted for surgery and were required to meet minimum starvation times as per SASA practice guidelines.

We excluded non-elective surgery, patients classified as American Society of Anesthesiologists (ASA) grade 3 or grade 4, patients with diabetes and those who declined to participate. We also excluded patients with multiple gestations due to the increased basal metabolic rate observed in these patients (up to 20% higher than that of singleton pregnancies).^17^ This may alter the risk of ketogenesis and metabolic acidosis and may have been a confounder.

Data were gathered over a period of 12 weeks, from 01 July 2023 to 22 September 2023. Each patient scheduled for an elective caesarean section received a research package before their procedure. This package contained an informed consent form and a fasting questionnaire, both of which were required to be completed before sampling.

Based on a pilot study at our institution, we estimated the incidence of abnormal acid/base status to be approximately 5%. To corroborate this finding with an alpha of 5% and a beta of 80%, a sample size of at least 73 patients was necessary. To enhance the study’s robustness and allow for missing data, we aimed to recruit approximately 100 patients. Consecutive eligible patients were recruited.

### Data collection

Starvation times were calculated in the pre-anaesthetic waiting area by the attending anaesthetist using a questionnaire. The questionnaire distinguished between the last intakes of fluids and solids. To calculate the total starvation time, the last intake of either solids or non-clear fluids was considered.

Questionnaires were completed by the patient, with assistance from the attending anaesthetist. Written questionnaires were available in isiZulu and English.

Blood samples were collected in the anaesthetic waiting area before entering the operation theatre, coinciding with the insertion of an intravenous to minimise any additional discomfort or risk to patients. Venous sampling for glucose is considered the gold standard compared with capillary sampling, and it also allowed us to obtain ketone and blood gas analyses without increasing patient discomfort.

Blood gas and ketone analyses were conducted within 15 min following sample collection. To avoid calibration discrepancies, all tests were performed using a single blood gas analyser and a single blood ketone monitor. All sampling and analysis were performed by sufficiently trained anaesthetists.

Before induction of anaesthesia, all results were communicated to the attending anaesthetist. Any abnormal findings, such as hypoglycaemia, were to be addressed by the attending anaesthetist. Subsequent anaesthesia was administered following the standards for obstetric anaesthesia at Harry Gwala Regional Hospital, in keeping with the Essential Steps in the Management of Obstetric Emergencies (ESMOE) anaesthesia guidelines designed for district and regional obstetric anaesthesia in South Africa. All trainee anaesthetists were supervised by an experienced anaesthetist, and specialist support was always available. Data were collected on paper-based forms and securely stored daily.

### Statistical analysis

Baseline characteristics were described as mean and standard deviation for parametric continuous data and as median and range for non-parametric data. Data distribution was evaluated graphically with histograms and scatter plots and numerically with skewness and kurtosis. Data comparisons were conducted using the Student’s *t*-test or Pearson’s correlation coefficient. We defined acidosis as a base excess less than −3 (normal range is between −2 and +2).^18^ Elevated ketones were defined as a blood ketone level of greater than 0.6 mmol/L. Hypoglycaemia in pregnancy is defined as plasma glucose ≤ 3.0 mmol/L, while levels < 3.9 mmol/L are considered low and warrant clinical attention, particularly in the perioperative obstetric setting.^19,20,21^ The normal range for base excess was defined as between +3 and −3.

### Ethical considerations

Ethical approval for the study was obtained from the Biomedical Research Ethics Committee of the University of KwaZulu-Natal and the Health Research Committee of the KwaZulu-Natal Department of Health. The ethical clearance number is BREC/00005504/2023. Written informed consent was obtained from all patients. Consent included discussions concerning blood sampling and the patient’s data that were used. Patient information was stored on a separate, password-protected database. Patients were identified by a unique number, and their identities remained anonymous.

We used the STROBE guidelines (Strengthening the Reporting of Observational Studies in Epidemiology) to report this observational study.^22^

## Results

A total of 111 patients were initially included. Four patients were excluded (two patients due to twin pregnancies and two patients due to incomplete forms). The final analysis included 107 patients. Baseline characteristics of these patients revealed a mean age of 31 years (standard deviation [s.d.] = 5.96), a median body mass index (BMI) of 30.8 kg/m^2^ (interquartile range [IQR] 7.8) and a median weight of 84 kg (IQR 17).

For the whole cohort, the mean starvation time was 13.2 h (s.d. = 3.2). The metabolic outcomes are shown in Table 1, stratified by fasting duration.

**TABLE 1:** Metabolic outcomes and fasting duration.

Metabolic parameter	Participant identifier	Fasting duration (hours)	Standard deviation	Confidence interval	*p*
Base excess ≤ -3	32	14.8	2.68	13.8–15.7	< 0.01
Base excess > -3	75	12.7	3.18	11.8–13.2	
Ketones ≥ 0.6	34	15.0	2.80	14.0–16.0	< 0.01
Ketones < 0.6	71	12.3	3.04	11.6–13.1	
Glucose < 3.9	67	13.8	3.10	13.1–14.6	0.01
Glucose ≥ 3.9	40	12.1	3.10	11.1–13.1	

Note: *p* values refer to comparisons of fasting duration for the listed metabolic parameters.

**FIGURE 1:**
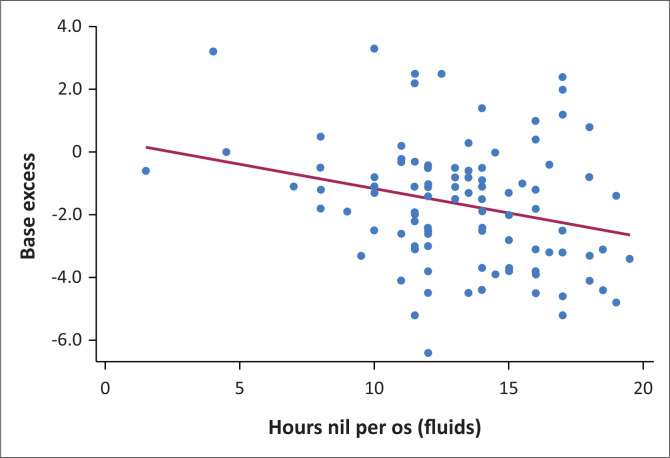
Scatter plot comparing fasted hours with the extent of acidosis (*N* = 107).

We first analysed base excess in relation to fasting duration, as depicted in the scatter plot (Figure 1). The analysis revealed a negative correlation (−0.252, *p* < 0.01), indicating that prolonged fasting corresponds with increasingly negative base excess values.

Among the 107 patients who participated, 32 met with the study definition of acidosis, characterised by a base excess < 3, representing 30% of the sample. The non-acidotic group displayed a shorter mean fasting duration of 12.75 h (s.d. = 3.18, 95% confidence interval [CI] 11.8–13.2), versus the acidotic group (mean fasting duration of 14.8 h [s.d. = 2.68], 95% CI 13.8–15.7); *p* < 0.01.

### Ketosis

**FIGURE 2:**
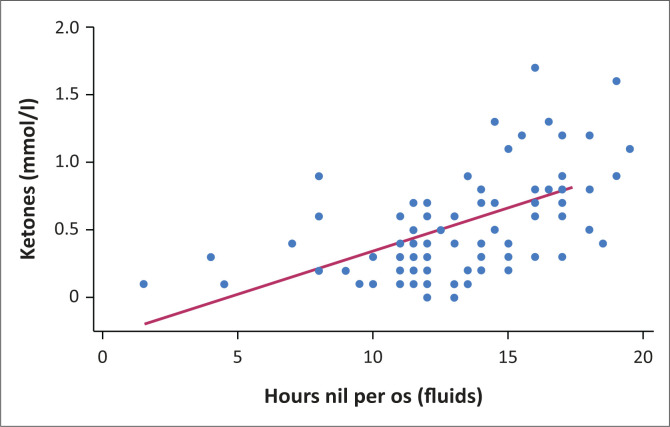
Scatter plot comparing hours *nil per os* with ketone levels (*N* = 107).

In this study, 32% of patients exhibited elevated ketone levels (> 0.6 mmol/L). Figure 2 presents a scatter plot comparing ketone levels with fasting duration. A positive correlation of 0.51 was found (*p* < 0.01).

The mean fasting time for patients with low/normal ketone levels was 12.3 h (s.d. = 3.04, 95% CI 11.6–13.1), while those with elevated ketones had a mean fasting time of 15.0 h (s.d. = 2.8, 95% CI 14.0–16.0); *p* < 0.01. This demonstrates the association between longer fasting periods and increased ketone levels (markedly worse after 12–14 h of starvation).

### Hypoglycaemia

We further investigated the relationship between fasting duration and glucose levels among patients. In total, 62 of 107 patients (62.6%) matched the definition of hypoglycaemia (blood glucose < 3.9 mmol/L). The correlation coefficient was −0.335 (*p* = 0.01), indicating a significant negative correlation between fasting duration and glucose levels. In the non-hypoglycaemic group, the mean fasting duration was 12.1 h (s.d.=3.1, 95% CI 11.1–13.1), versus 13.8 h (s.d. = 3.1, 95% CI 13.1–14.6) in the hypoglycaemic group; *p* < 0.01. This indicates that prolonged fasting is closely associated with hypoglycaemia in these patients. Patients found to be hypoglycaemic were managed appropriately, and blood glucose was corrected preoperatively.

## Discussion

Our study identified elevated levels of acidosis and ketosis in patients undergoing elective caesarean sections after prolonged preoperative fasting periods. Additionally, 63% of these patients exhibited hypoglycaemia, which correlated with their fasting duration. Existing literature on acid–base disturbances and ketosis in obstetric patients is scarce and primarily anecdotal in nature. The presence of acidosis and hypoglycaemia could pose serious perioperative risks, and our research highlights its prevalence.

The 2022 SASA practice guidelines recommend a fasting period of 6 h for solids and 2 h for liquids to minimise the risk of aspiration.^23^ Our study confirms a marked deviation from this, in keeping with other studies.^8^ The extended fasting periods can be attributed to a variety of systemic challenges, including strict ward mealtime schedules, limited availability of nursing staff and unpredictable surgical schedules.

This study confirmed that prolonged starvation may be associated with ketosis, acidosis and hypoglycaemia. A significant factor influencing these prolonged starvation periods is the lack of a dedicated theatre for elective caesarean sections at the participating hospital, leading to emergency procedures taking preference. This prioritisation results in patients being kept *nil per os* (NPO) until an operating slot becomes available, thereby prolonging fasting. This practice is intended to avoid the underutilisation of theatre time and prevent the cancellation of scheduled operations. However, it compromises adherence to fasting guidelines and potentially impacts patient safety and comfort. The hospital’s operational constraints necessitate a balance between optimising theatre utilisation and minimising preoperative fasting times. Addressing these issues may require implementing more flexible ward scheduling, enhancing resource allocation and considering strategic planning to mitigate theatre congestion. Engaging stakeholders to explore innovative solutions could help align the operative scheduling practices more closely with evidence-based guidelines, ensuring both optimal patient care and resource efficiency.

Although this challenge is described within the context of the participating hospital, similar constraints are encountered in many public-sector hospitals across South Africa, as seen in the study by Morgan et al.^8^ where limited theatre availability and high emergency surgical burdens frequently disrupted elective scheduling. These systemic pressures often result in prolonged preoperative fasting for obstetric patients. Therefore, the findings from this setting may reflect broader operational challenges within resource-constrained healthcare environments and highlight the need for national attention to perioperative fasting practices and theatre resource allocation.

Previous investigations into acidosis, encompassing case studies and series, typically report a starvation duration of 24–48 h.^4,9,11,12,13,14,15,16^ However, such durations have frequently resulted in patients presenting with critical illness, characterised by severe acidosis that necessitates intervention, sometimes requiring intensive care unit admission. This study demonstrates that 30% of patients developed acidosis after a mean starvation period of 14.8 h, with base excess values ranging from −3.1 to −6.4.

In the study conducted by Morgan et al. in 2021, the researchers examined the incidence of hypoglycaemia among patients undergoing caesarean sections.^8^ Despite attempted adherence to NPO guidelines, the subjects in this study experienced a similar duration of preoperative fasting to our cohort. They revealed a 39% incidence of hypoglycaemia within the tested population. In parallel, our trial adopted the same hypoglycaemia criteria but demonstrated a markedly higher rate of incidence of 62%. Furthermore, both studies similarly identified a statistically significant correlation between the length of the starvation period and the occurrence of hypoglycaemia. These findings highlight the critical need to re-evaluate current preoperative fasting guidelines to potentially mitigate the risk of hypoglycaemia and associated complications in patients undergoing caesarean section.

### Strengths and limitations

Available data on this topic is currently limited. One of the major strengths of this study is that it identifies the correlation between prolonged starvation and the presence of acidosis, ketosis and hypoglycaemia. The findings are clinically relevant and potentially preventable, highlighting opportunities to improve perioperative care for obstetric patients.

Limitations of our study included its single-centre design. This is a cross-sectional descriptive study that does not infer causation. We restricted our patients to those scheduled for elective surgery, as we wished to exclude the confounding metabolic requirements linked to labour. We also included only ASA 1 and 2 patients in this trial to limit the additional effects of comorbidities and medications on metabolic status. Patients presenting for emergency caesarean section, patients who are not ASA 1 or 2 and patients with multiple pregnancies might benefit from further research. We also did not question women about maternal satisfaction and the degree of hunger, which could have provided important qualitative data.

### Recommendations

Our study suggests that corrective measures should be implemented during the preoperative period. Centres providing obstetric care should review their starvation periods before operation and implement quality improvement protocols where applicable.

Sip-til-Send is an initiative aimed at reducing prolonged preoperative fasting by challenging traditional fasting guidelines that require 6 h for solids and 2 h for clear fluids before surgery.^24^ The protocol allows patients to consume clear fluids, such as water or tea without milk, until transfer to the operation theatre complex, and in some cases includes caloric beverages for non-diabetic patients. Studies have demonstrated substantial reductions in fasting duration, from approximately 12 h to 1.5 h.^25^ In South Africa, where obstetric theatres often face scheduling pressures and emergency case interruptions, this approach may improve perioperative care and maternal satisfaction without increasing aspiration risk.^26^

## Conclusion

Our study showed that patients being fasted for elective caesarean section developed acidosis, ketosis and hypoglycaemia in proportion to the duration of the fasting period. This study indicates that there is still room for marked improvement in the quality of care provided to obstetric patients during the perioperative period. Future research should aim to identify methods to reduce fasting durations in contexts such as those described through quality improvement initiatives and to measure the impact on both foetal and maternal morbidity.
